# Mannan-BAM, TLR Ligands, Anti-CD40 Antibody (MBTA) Vaccine Immunotherapy: A Review of Current Evidence and Applications in Glioblastoma

**DOI:** 10.3390/ijms22073455

**Published:** 2021-03-26

**Authors:** Pashayar P. Lookian, David Zhao, Rogelio Medina, Herui Wang, Jan Zenka, Mark R. Gilbert, Karel Pacak, Zhengping Zhuang

**Affiliations:** 1 Neuro-Oncology Branch, National Cancer Institute, National Institutes of Health, Bethesda, MD 20892, USA; pasha.lookian@nih.gov (P.P.L.); dyz@georgetown.edu (D.Z.); rogelio.medina@nih.gov (R.M.); herui.wang@nih.gov (H.W.); mark.gilbert@nih.gov (M.R.G.); 2 Department of Neurosurgery, University of Nebraska Medical Center, Omaha, NE 68198, USA; 3 Department of Neurosurgery, Medstar Georgetown Hospital, Washington, DC 20007, USA; 4 Department of Medical Biology, University of South Bohemia Cheske Budejovice 37005, Czech Republic; jan.zenka@nih.gov; 5 Section on Medical Neuroendocrinology NICHD/DIR, National Institutes of Health, Bethesda, MD 20892, USA; karel@mail.nih.gov

**Keywords:** mannan-BAM, Toll-like receptor ligands, anti-CD40, metastatic, immunotherapy, innate immunity, adaptive immunity, Toll-like receptor, pathogen-associated molecular patterns, neutrophil, T cell, glioblastoma

## Abstract

The foundation of precision immunotherapy in oncology is rooted in computational biology and patient-derived sample sequencing to enrich for and target immunogenic epitopes. Discovery of these tumor-specific epitopes through tumor sequencing has revolutionized patient outcomes in many types of cancers that were previously untreatable. However, these therapeutic successes are far from universal, especially with cancers that carry high intratumoral heterogeneity such as glioblastoma (GBM). Herein, we present the technical aspects of Mannan-BAM, TLR Ligands, Anti-CD40 Antibody (MBTA) vaccine immunotherapy, an investigational therapeutic that potentially circumvents the need for in silico tumor-neoantigen enrichment. We then review the most promising GBM vaccination strategies to contextualize the MBTA vaccine. By reviewing current evidence using translational tumor models supporting MBTA vaccination, we evaluate the underlying principles that validate its clinical applicability. Finally, we showcase the translational potential of MBTA vaccination as a potential immunotherapy in GBM, along with established surgical and immunologic cancer treatment paradigms.

## 1. Introduction

**Figure 1:**
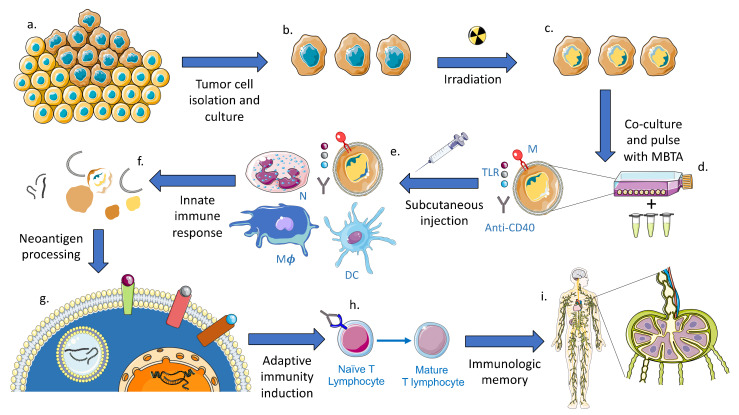
Shown is the mechanism of action for MBTA vaccine therapy. (**a**,**b**) Patient tumor tissue is isolated and expanded through cell culture for irridation (**c**). The irradiated tumor cells are then (**d**) co-cultured and pulsed with Mannan-BAM (M), TLR Ligands (TLR), and Anti-CD40 Antibody (MBTA). Following MBTA pulse, the co-cultured, irradiated tumor cells are injected subcutaneously into a remote peripheral site (**e**). A local innate inflammatory respond is initiated and results in the recruitment of innate effector cells, such as neutrophils (N), macrophages (M*ϕ*), and dendritic cells (DC). Formation of the inflammasome and destruction of the irradiated tumor cell (**f**) leads to release of native immunogenic neoantigens for (**g**) processing by antigen presenting cells (APCs). This leads to induction of adaptive immunity (**h**) and generation of memory anti-tumor response (**i**).

Mounting evidence has demonstrated the efficacy of mannan-BAM, Toll-like receptor (TLR) ligands, and anti-CD40 monoclonal antibodies (MBTA) vaccines in generating an anti-tumor response in solid tumors [1,2]. This vaccine strategy leverages various immunogenic components that stimulate both the innate and adaptive immune responses such as pathogen associated molecular patterns (PAMPs), TLR ligands, and antigen presenting cell (APC) costimulatory proteins (Figure 1).

Mannan is a polysaccharide derived from *Saccharomyces cerevisiae* that is anchored to the cell membrane via conjugation to Biocompatible Anchor for Cell Membrane (BAM), serving as a cell membrane anchored PAMP to induce cell phagocytosis and complement activation via the complement lectin pathway [1,2,3,4,5,6,7]. The TLR ligands lipoteichoic acid (LTA), polyinosinic-polycytidylic acid (poly-IC), and resiquimod (R-848) serve to augment the innate immune response via induction of several inflammatory cascades [8,9,10,11,12,13,14]. The highly immunogenic LTA, derived from *Bacillus subtilis*, increases TNFα secretion by activating the TLR2-mediated inflammatory pathway [8,9]. Poly-IC is a synthetic analog of viral double-stranded RNA that activates TLR3-mediated signaling, thereby activating APCs and altering tumor-associated macrophages to adopt an immunologically active phenotype [8,10,11]. R-848 is a synthetic analog of viral single-stranded RNA that activates the TLR7-mediated signaling, leading to innate immune cell activation and stimulation of Th1 cell-mediated immunity [12,13]. Finally, this immunotherapeutic strategy includes anti-CD40 monoclonal antibodies to activate CD4+ T lymphocytes via binding of CD40L, licensing dendritic cells in induction of the adaptive immune response [14,15,16].

Previous investigations using MTBA vaccine therapy reported efficacy in achieving a sustained anti-tumor response but were limited by the need for intratumoral MBTA injection. This delivery method, directly into the tumor in situ, precluded MBTA treatment in patients with tumors in eloquent areas susceptible to secondary inflammatory damage or mass effect [17,18,19,20,21,22]. In our prior MBTA study, we demonstrated in a preclinical mouse model of colon carcinoma that subcutaneous vaccine delivery of irradiated whole tumor pulsed with MBTA resulted in significantly decreased tumor volumes and increased overall survival when compared to saline or irradiated whole tumor cell vaccine alone [23].

By pulsing MBTA with resected autologous whole tumor cells that underwent sublethal irradiation, the vaccine constituents are assembled in vitro with intact whole tumor cells that have been inactivated as a result of radiation-induced (1) cell cycle arrest, (2) direct induction of apoptosis, (3) and mitotic catastrophe [24]. Subsequent subcutaneous injection of inactivated tumor cells and MBTA into a peripheral site allows for tumor-specific neoantigen processing in vivo through endogenous pathways that activate the innate immune system and result in stimulation of a sustained adaptive immune response [23].

Herein, we review the current state of the most promising GBM-specific vaccine immunotherapies. We then examine the evidence supporting an immune-based vaccine therapy that utilizes mannan-BAM, TLR Ligands, anti-CD40 antibody (MBTA) to induce both an innate and adaptive immune response to tumor cells as a potential treatment specifically for GBM.

## 2. Current GBM Vaccine Immunotherapy

An estimated 80,000 new primary tumors of the brain and central nervous system are diagnosed each year in the United States, with increasing incidence over the recent years [25]. Despite its low overall incidence, glioblastoma (GBM), defined as a grade IV astrocytoma without IDH mutation, remains as the most deadly primary brain neoplasm despite major medical advances that have occurred in the fields of oncology, radiation oncology, and neurosurgery. To date, the standard of care includes maximal safe surgical resection, followed by concurrent radiation and temozolomide chemotherapy, and adjuvant temozolomide. Despite this regimen, the average prognosis for patients with GBM is 18–23 months with an estimated 2–4% five-year survival rate [26].

Given the need for improved therapeutic intervention and clinical outcomes, better understanding at the molecular level of GBM and the surrounding tumor microenvironment has become an increased focus of investigation. Intratumoral heterogeneity and plasticity resulting from different molecular aberrations and in response to single modality drug therapy have become the greatest obstacle in finding better treatments for patients with GBM. The alterations in the tumor microenvironment due to the primary pathological changes in the tumor parenchyma increasingly demonstrate a relationship with disease progression, adding an additional layer of challenge in discovering effective therapy. The genetic heterogeneity of GBM, mutational adaptability, and selective interactions with the tumor microenvironment have led to great interest in developing tumor-specific immune-based therapies capable of generating a targeted response against the heterogenous, adaptable antigens of GBM.

Generally, vaccination against cancer has several advantages when compared to the standard immunotherapies. Vaccination leverages the host’s own immune system to actively target specific epitopes of the tumor, allowing for tumor recognition and destruction, which is followed by a sustained memory response. Various vaccine strategies have been developed by modifying the constitutive vaccine components and altering method of delivery, in combination with adjuvant or neo-adjuvant therapy [27]. GBM vaccine investigations are generally targeted at tumor-specific peptides or exposing dendritic cells (DCs) to tumor components to induce immunostimulatory responses. Recently, there have also been clinical trials with vaccines aimed at heat shock protein 96 (HSP96), due to its high expression in GBM tumors, with relative success. However, we do not review this strategy in depth as further studies are required to overcome limitations in vaccine production and inhibitory cytokines in the tumor microenvironment [28]. A list of clinical trials using peptide vaccines or DC-based vaccines are included in Table 1 and Table 2, respectively. We believe that the table is formatted similarly to standards of reporting clinical trials in the field of neurooncology.

To date, GBM peptide vaccine design has been directed at generating a vaccine against Epidermal growth factor receptor (EGFR). Approximately 20–30% of GBM patients have tumors that harbor an in-frame deletion of exons 2–7 coding for an isoform of EGFR, named EGFR variant III (EGFRvIII), which has been used as the target antigen in pre-clinical models and clinical trials. However, there has been a lack of translational results from the pre-clinical animal models to clinical trials with EGFRvIII peptide vaccines, which have not been shown to have a substantial clinical benefit. This can be attributed to the observation that intratumoral expression of the variant EGFRvIII antigen is not consistent among tumor cells [27]. Moreover, it has been demonstrated that EGFRvIII expression is lost in approximately 30–50% of cases of GBM recurrence in patients with a prior established EGFRvIII mutation. Collectively, these barriers lead to tumor cell selection, propagation of EGFRvIII-negative cells, and generation of an adaptive immune memory response to a transient tumor antigen [27].

There has been an increase in the use of DC-based vaccines, as pre-clinical models have shown effective methodologies of exposing antigen presenting cells (APCs) to various tumor cell components in order to induce a stable immune anti-tumor response. Investigations in pre-clinical models and phase I clinical trials have been done by pulsing DCs with proteins, tumor lysates, RNA, and other cellular components [27,29]. Despite the encouraging results in early studies showing therapeutic efficacy and safety of DC-based vaccines, the data available are currently limited, even with on-going and completed phase II/III trials. As of this writing, one ongoing phase III clinical trial (NCT00045968) has been conducted in primary GBM patients that were randomized to receive either TMZ and an autologous tumor lysate-pulsed DC vaccine or TMZ plus placebo [27,29]. Early results of this ongoing clinical trial have shown an 8-month survival benefit; however, there are limitations to interpretations of this data such as lack of IDH mutation status subgroup analysis, current status of study, and high crossover fraction due to use of the vaccine upon tumor recurrence in the study sample.

Further, there are significant limitations in the use of immunotherapy as it relates to GBM that span across all potential immune-based therapeutic modalities, including vaccination. At the molecular level, GBM exhibits intratumoral heterogeneity and low immunogenicity. The accumulation of molecular alterations conferring increased survivability is largely due to the selective pressures generated by competition for metabolic resources [30,31]. The tumor microenvironment generated by this process enriches for cells with increased survivability mutations, conferring resistance to both the immune system and drug therapy. This has led to the recognition that GBM induces an immunosuppressive tumor microenvironment as tumor cells harboring immunologically cold epitopes [27,28,29,30,31]. Systemically, recent studies have shown that GBM affects the immune phenotype in the peripheral circulation and the central nervous system (CNS), which has been traditionally considered to be an immune privileged space [27,28,29,30,31]. Even more limiting is the reliance on tumor-specific epitope sequencing that requires a tumor specimen from the patient, which is then sequenced and used to derive tumor-specific antigens in silico. While this process can enrich for specific epitopes that are expressed by the tumor, the likelihood of that epitope being both tumor-specific and ubiquitously expressed throughout the tumor to serve as a reliable target for the immune system is low [32]. This has been the greatest limiting factor for vaccination strategies based on identifying immunogenic epitopes because they are generally targeted at a single peptide.

As a result, further investigation into vaccination strategies that subvert these barriers has become an area of increased focus in cancer immunology. Ideally, a vaccination methodology that is capable of inducing a specific adaptive response to a wide variety of immunogenic epitopes, shifts the tumor microenvironment into a less tumor suppressive phenotype, and enhances the systemic immune anti-neoplastic response will be discovered through these ongoing efforts. In combination with previous work on MBTA immunotherapy, we highlight the potential of an MBTA vaccine in GBM as it overcomes the aforementioned challenges of effective recognition of tumor specific neoantigens and the induction of an anti-tumor immunophenotype within the tumor microenvironment.

## 3. Preclinical Investigations of MBTA Immunotherapy in Oncology

Several studies have established that constituents of MBTA immunotherapy result in a substantial response in murine subcutaneous tumors including melanoma, sarcoma, pancreatic adenocarcinoma, and pheochromocytoma [1,2,4,7,23,33]. One of these studies demonstrated the significant reduction in tumor volume and increased overall survival in a mouse model of metastatic pheochromocytoma treated with intratumoral mannan-BAM and TLR ligands (MBT) injection [1,2]. The experimental mice in this study showed a robust innate and adaptive immune response that resulted in long-term tumor-specific memory upon rechallenge [1,2].

The pheochromocytoma mouse model in the study was established by subcutaneous or intravenous injection of luciferase-expressing mouse tumor tissue (MTT) pheochromocytoma cells. Tumor size and presence of metastatic organ, particularly liver, lesions were measured in addition to urine catecholamine levels. Intratumoral treatment with MBT compared to PBS (control) resulted in significant stabilization of subcutaneous tumor size and increased overall survival (median 50-days compared to 16-days in the control group) [1,2].

The experiment was repeated using B6 *scid* mice lacking functional T and B cells to evaluate the role of innate immunity. All mice were sacrificed at 30 days and tumors were harvested for additional analysis. Again, it was demonstrated that intratumoral MBT treatment led to statistically significant smaller tumor volume compared to control. In addition, immunohistochemical analysis revealed higher levels of CD45+ tumor-infiltrating leukocytes (TILs) in the MBT treatment group compared to control [1,2].

Flow cytometry analysis of TILs in immunocompetent mice revealed that tumors treated with MBT demonstrated increased levels of CD4+ and CD8+ cells compared to control. Upon histological analysis, extensive necrotic areas of tumor in MBT-treated samples were INF-γ to interleukin 10 level ratio (INF-γ/IL-10) revealed a Th1 shift in the tumor microenvironment of MBT-treated tumors [1,2].

Further experiments combining MBT with anti-CD40 (MBTA) demonstrated a beneficial effect, leading to increased survival when compared to MBT-treated mice. There were five mice in the MBTA treatment group that were found to completely eliminate subcutaneous tumor, and re-challenge with MTT pheochromocytoma cell injection resulted in detectable tumor development, which was followed by complete tumor elimination. Collectively, these results suggest that MBTA therapy activates an innate and adaptive immune response leading to anti-tumor effects.

MBTA was then evaluated on a metastatic pheochromocytoma model, established by concurrent subcutaneous and tail-vein injections of luciferase-expressing tumor cells. Metastasis, mainly hepatic, was confirmed with an in vivo bioluminescence assay [1,2].

Intratumoral treatment of MBTA into subcutaneous tumor resulted in a lower bioluminescence intensity in the primary subcutaneous injection site as well as the metastatic lesions, with significantly increased overall survival in the MBTA treatment group compared to the PBS-treated control group. These studies were replicated in CD4+ and CD8+ depleted mice which demonstrated the importance of T cells in MBTA therapy, with CD4+ and CD8+ depleted treatment groups showing comparable metastatic bioluminescence intensity and similar overall survival to control mice [1,2].

Further, in our previous MBTA study using a murine colon carcinoma CT26 cell line, we demonstrated that intratumoral injection of MBTA as well as subcutaneous delivery of a vaccine consisting of irradiated autologous, whole tumor cell pulsed with MBTA could induce a robust tumor-specific adaptive immune response [23]. Primary and metastatic representative tumors were established by CT26 tumor cell inoculation into the right and left flank, respectively [23]. Intratumoral injection of MBTA versus saline into the primary right flank tumor was administered according to the treatment schedule. Following treatment, MBTA-injected mice demonstrated significantly decreased primary right flank as well as metastatic left flank tumor volumes. Furthermore, MBTA-treated mice demonstrated improved overall survival compared to control saline-treated mice [23].

Therapeutic efficacy of MBTA was shown to be dependent on T-cell activity in the context of an adaptive immune response. CD4+ and CD8+ T-cell depleted mice were subjected to the same intratumoral MBTA treatment schedule. It was demonstrated that primary tumor volume in CD8+ T-cell depleted mice was significantly larger when compared to tumor volume of non-T-cell depleted mice. In addition, non-T-cell depleted mice demonstrated increased median survival when compared to their T-cell depleted counterparts [23].

Primary and metastatic tumors were harvested and subjected to immunophenotyping (IP) analyses to characterize the immune profile of the tumor microenvironment in response to MBTA treatment versus saline injection (control). These studies demonstrated acute trafficking of neutrophils and APCs to MBTA-treated primary tumor, promoting phagocytosis and antigen processing for subsequent development of an adaptive immune response. Further cytokine secretion analyses of CD8+ T-cell populations taken from the metastatic tumor site in MBTA-treated mice demonstrated a significant increase in INF-γ and TNFα. These results suggest that injection of MBTA into primary tumor sites can modulate CD8+ T-cell activation within metastatic tumor sites [23].

These studies demonstrated that subcutaneous vaccine delivery of irradiated whole tumor pulsed with MBTA to tumor-bearing mice resulted in smaller median tumor volumes and significantly increased survival when compared to saline or irradiated whole tumor cell vaccine alone. After 50 days, mice that achieved a complete regression of tumor after subcutaneous treatment with irradiated CT26-MBTA vaccine were re-challenged with reinoculation of CT26 cells at a naïve site, and none (0/4) of these mice displayed evidence of tumor growth. This confirmed the generation of a potent anti-tumor immune response with antigen-specific long-term memory [23].

Intracranial re-challenge studies underscored the development of robust immunological memory to prevent intracranial tumor growth as well. The same mice that achieved complete regression of tumor after subcutaneous treatment with irradiated CT26-MBTA vaccine were subject to stereotactic implantation of CT26-Luc cells in the frontal lobe. While all (4/4) control mice developed intracranial tumor and died within 30 days of implantation, none (0/4) of the mice vaccinated with irradiated CT26-MBTA demonstrated evidence of intracranial tumor, measured via bioluminescence [23]. Taken together, the results of these studies demonstrate that the MBTA immunotherapeutic strategy, tested in several different types of mouse tumor models, is unique in the manner by which initial activation of innate immunity induces activation of long-lasting adaptive immunity.

## 4. Future Application of MBTA Vaccine Therapy in GBM

As previously established, classical strategies used in oncology, where tumor tissue can be obtained from a patient, homogenized, sequenced, and used to derive tumor-specific antigens, are less commonly used in neuro-oncology. Despite the pragmatic obstacles, the aforementioned low intratumoral immunogenicity and high intratumoral genetic heterogeneity in GBM have consistently undermined immunotherapeutic strategies that rely on tumor antigen target identification through these methods [27,30,32]. Leveraging these tumor-specific epitopes has led to improved patient outcomes in many types of cancers that were previously untreatable. However, GBM tumor cells have variable tumor mutational burden (TMB), which is inversely correlated with overall survival, and carry a high aptitude for mutation [34]. As a result, in silico enrichment of neoantigens with high enough immunogenicity capable of eliciting a targeted, sustained anti-tumor response is, unfortunately, unlikely to discover any additional immunotherapeutic targets that are broadly clinically applicable. This is compounded by the high degree of intratumoral mutational heterogeneity within a single GBM tumor. Meaning that, despite ongoing sequencing efforts to determine the idiosyncratic genetic make-up of GBM coupled with immense knowledge gained from data bases such as The Cancer Genome Atlas (TCGA), there is no guarantee that an individual GBM tumor would respond equivocally to a targeted immunotherapy due to lack of conservation of the immunogenic epitope ubiquitously throughout the tumor.

In order to circumvent these obstacles, we hypothesized that combining MBTA with whole cell irradiation will allow us to induce an innate immune phenotype in vitro that could then be used to condition an adaptive immune response in vivo. By leveraging MBTA vaccine treatment with tumor cell co-culture and irradiation, we have previously established the ability to induce a pro-inflammatory innate response that transitions into a CD8+ cytotoxic T lymphocyte response in other murine cancer models [23]. Further, this vaccination strategy accounts for the lack of immunogenic antigens and intratumoral genetic heterogeneity in GBM because the immunostimulatory conjugates allow for in vitro anchoring of the mannan-BAM, and for future induction of TLR2, TLR3, and TLR7-mediated signaling through adjuvants, that leads to in vivo innate immune cell activation and stimulation of Th1 cell-mediated immunity, which are further enhanced by tumor cell irradiation. Subsequent injection of MBTA pulsed with the irradiated whole tumor cells into a peripheral site induces a controlled, local inflammatory reaction that allows for the remaining components of MBTA to work in concert with the host immune system to destroy the irradiated tumor cells, releasing antigens, and activating APCs for antigen processing and presentation.

Specifically, MBTA mixed with irradiated tumor cells are subcutaneously injected into a peripheral site where the highly immunogenic components of MBTA therapy induce a localized inflammatory reaction in the dermis. The resulting inflammasome disrupts the integrity of the irradiated tumor cells by subjecting them to the endogenous processes of the innate immune response via recruitment of neutrophils, macrophages, and dendritic cells as shown in our previous work [23]. The result is tumor-specific neoantigen processing by APCs without the need for sequencing. This method allows for the induction of adaptive immune response by allowing the epitopes to be self-selected by the immune system using the canonical pathway used to identify foreign proteins. The in vitro arm of this combinatorial approach potentiates an innate inflammatory response for tumor cell destruction, followed by the in vivo induction of that response and release of immunogenic antigens directly from the tumor cells, generating high fidelity, specific tumor epitopes that are likely to be unaccounted for by traditional sequencing-based techniques. Therein, the mechanism by which MBTA vaccine therapy generates immunotherapeutic targets represents a unique vaccination strategy that is not directed at a single epitope, such as those targeted at mutated EGFR, rather it creates multiple tumor-specific targets by allowing the innate immune system and APCs to select for antigenic targets through endogenous processing and signaling cascades.

In addition, we also previously reported that in mouse models of metastatic disease, MBTA treatment significantly increased the quantity of CD8+ cytotoxic T lymphocytes, with a sustained response on second challenge with subcutaneously injected tumor cells [23]. We demonstrated with this work that MBTA vaccination initially generated a predominantly neutrophilic immunophenotype on day 10 post-MBTA vaccine treatment that transitioned into a primarily CD8+ cytotoxic T lymphocytes immunophenotype by day 16. These results indicate that MBTA vaccine therapy, when implemented in an approach as we present here, has the potential to generate an endogenous innate inflammatory response directed at irradiated tumor cells conjugated to MBTA, which generates numerous immunogenic epitopes that stimulate APCs and results in the generation of a stable, specific, and adaptive CD8+ cytotoxic T lymphocyte anti-neoplastic response. These findings suggest that MBTA vaccine therapy has potential utility in reversing the systemic immunosuppressive and tumor microenvironment effects seen in patients with GBM [29,30,31]. Moreover, our MBTA vaccine shows promising efficacy in treating GL261-Luc cells in our preliminary studies [33].

Despite these encouraging proof-of-concept studies, the utility of MBTA vaccine immunotherapy in GBM requires additional investigation for several reasons. High grade gliomas such as GBM have a propensity to infiltrate surrounding normal brain tissue because of their uncontrolled cell proliferation, apoptotic resistance, and genomic instability [35]. This presents a significant challenge in immunotherapy because the diffuse nature of infiltration can limit the ability of the immune system to produce a tumor-specific response without off-target damage to the surrounding parenchyma. Further, the limited volume of the cranial vault is unamenable to the osmolar changes that would accompany the induced immunologic and metabolic pathways, leading to an edematous state with a significant risk for adverse outcomes such as a systemic inflammatory syndrome or herniation. Given the mechanism by which MBTA vaccine immunotherapy leverages the innate immune system to induce a memory response, the terminal effector adaptive immune cells that would prevent recurrence or disease progression are T lymphocytes. Therefore, it is likely that the toxicities noted with other T lymphocyte-based therapies such as CAR-T cells, which have been associated with immune effector cell-associated neurologic syndrome (ICANS) or cytokine release syndrome (CRS), could result with MBTA vaccination in GBM and other CNS tumors [36,37].

Moreover, cell culture and murine models of GBM have significant variation from endogenous cases of GBM in humans, and thus the translational implications of studies utilizing these modalities continue to be a limiting factor in translational neuro-oncological research. Thus, conclusions regarding the migration of peripherally induced immune cells into the CNS cannot be drawn with significant translational accuracy. Finally, injection of irradiated whole tumor cells and the immunogenic constituents of MBTA into a peripheral subcutaneous injection site presents obvious pragmatic challenges with regard to clinical applicability. Despite the well-documented effects of ionizing radiation on tumor cells, injection of irradiated GBM tumor cells into a remote site still carriers the risk of iatrogenic tumor seeding and is unlikely to be accepted as a viable treatment modality by either patients or their physicians. However, the need for improvement in therapeutic intervention and clinical outcomes in patients with GBM may spurn these limitations and encourage future pre-clinical studies to evaluate the translational immunotherapeutic potential of MBTA vaccine therapy.

## 5. Conclusions

Herein, we present the emerging immunotherapeutic potential, technical aspects and the foundational investigations that have established MBTA vaccines as a potential therapeutic intervention for many types of primary cancers and metastatic disease. We also review the current landscape of the field of vaccine immunotherapy for GBM, including ongoing clinical trials and current limitations of the most widely utilized vaccine strategies. Given the molecular heterogeneity of GBM, poor response to drug therapy, and interactions with the tumor microenvironment, we envision MBTA immunotherapy to play a distinct role as a future therapy in GBM as it allows for recognition of tumor-specific neoantigens, simultaneous response to multiple immunotherapeutic targets, and the induction of an anti-tumor immunophenotype within the tumor microenvironment. With future studies, we believe that MBTA immunotherapy, in combination with established neurosurgical, chemotherapeutic, and radiotherapeutic interventions, may lead to the paradigm shift desperately needed in the continued efforts to improve patient outcomes in GBM.

## Figures and Tables

**Table 1:** Clinical glioblastoma peptide vaccine trials. Autologous Peptides (Peptides—Auto.); Progression Free Survival (PFS); Overall Survival (OS).

Clinical Trial	Phase	Target	Number of Patients	Endpoint	Outcome
ACTIVATe NCT00643097 20921459	II	EGFRvIII	18	PFS at 6 months	94% v. 59%
ACT-II 21149254	II	EGFRvIII	22	OS	23.6 v. 15.0 mon
ACT-III 25586468	II	EGFRvIII	65	PFS at 5.5 months	66% v. 45%
ACT-IV NCT01480479 28844499	III	EGFRvIII	371	OS	No significant difference
ReACT NCT01498328	II	EGFRvIII	33	PFS at 6 months	27% v. 11%
HSPPC-96 NCT00905060 II	II	Peptides - Auto.	46	OS	24.0 months
HSPPC-96 NCT00293423 24335700	II	Peptides - Auto.	41	OS at 6 months	90.2%
HSPPC-96 NCT01814813	II	Peptides - Auto.	30	OS	No significant difference
ITK-1 UMIN000006970 30500939	III	Tumor Associated Antigens	58	OS	No significant difference
SL-701 NCT02078648	II	Tumor Associated Antigens	74	OS at 12 months	43%
IMA-950 NCT01920191 30753611	II	Tumor Associated Antigens	16	OS	19.0 months

**Table 2:** Clinical glioblastoma dendritic cell vaccine trials. Tumor Lysate (TL); Progression Free Survival (PFS); Overall Survival (OS).

Clinical Trial	Phase	Antigenic Target	Number of Patients	Endpoint	Outcome
DENDR1 29632727	I/II	TL	24	PFS at 12 months	41%
DENDR2 NCT02820584 - 1	I/II	TL	12	OS	7.4 months
DENDR2 NCT02820584 - 2	I/II	TL	8	OS	9.3 months
DEND/GM NCT01006044 28499389	II	TL	31	PFS	12.7 months
DCVax-L NCT00045968 29843811	III	TL	331	OS	23.1 months
GBM-Vax NCT01213407 30301187	II	TL	34	PFS at 12 months	No significant difference
NCT00576537 18632651	I/II	TL	34	OS	Responders: 21.0 months Non-responders: 14.0 months
NCT00323115 21499132	II	TL	10	OS	28.0 months
NCT03879512 30054667	II	TL	11	OS at 6 months	100%
ICT-107 NCT01280552 31320597	II	Tumor Associated Antigens	81	OS	17.0 months
NCT02772094 21715171	I/II	Irradiated Tumor Cells	16	OS at 12 months	17.0 months
NCT01567202 30159779	I/II	Glioma Stem Cell Associated Antigens	22	OS	7.7 months
DC-CAST-GBM NCT00846456 23817721	I/II	Tumor mRNA	7		25.0 months
